# Reimagining Antimicrobial Drug Delivery: Spatiotemporally Adaptive Therapeutics for Biofilm-Associated Infections and Antimicrobial Resistance

**DOI:** 10.3390/pharmaceutics18101202

**Published:** 2026-09-22

**Authors:** Eman Marzouk, Mahmoud Jaber

**Affiliations:** Department of Public Health, College of Applied Medical Sciences, Qassim University, P.O. Box 6666, Buraydah 51452, Saudi Arabia; mf.jaber@qu.edu.sa

**Keywords:** antimicrobial drug delivery, spatiotemporal drug delivery, biofilm-associated infections, antibiotic resistance, infection-responsive delivery, controlled drug release, localized antimicrobial delivery, multimodal antimicrobial therapy, advanced drug delivery systems

## Abstract

Antimicrobial therapy may fail even when an agent is intrinsically active if effective drug exposure is not achieved at the site of infection at the appropriate time. This challenge is particularly important in biofilm-associated infections and infections caused by drug-resistant pathogens, where biological barriers, spatial heterogeneity, and changing microbial states can limit treatment effectiveness. This narrative review examines advanced antimicrobial delivery from a problem-oriented perspective, focusing on the therapeutic challenges that delivery systems are designed to address. It explores how antimicrobial exposure can be aligned with the location, biological state, and changing course of infection. The review examines the biological and pharmacological barriers that restrict antimicrobial activity and discusses strategies designed to improve drug access, retention, release, and local activity. Localized delivery, responsive and externally triggered systems, controlled and sequential delivery, and multimodal therapy are considered as implementation strategies within a common spatiotemporal framework. Particular attention is given to the relationship between formulation design, local drug exposure, biological response, and therapeutic outcome. The review further examines the factors that determine whether a technically promising system has a realistic clinical role, including safety, reproducibility, manufacturability, model relevance, and demonstrable benefits over simpler approaches. Future priorities are considered from a clinically defined perspective. Overall, the review argues that the value of advanced antimicrobial delivery lies not in carrier complexity alone, but in its capacity to provide a measurable and reproducible therapeutic advantage for a clearly defined infection setting.

## 1. Introduction

Antimicrobial resistance (AMR) is reducing the effectiveness of antibiotic treatment worldwide. In 2023, approximately one in six laboratory-confirmed bacterial infections causing common infections was resistant to antibiotic treatment, while resistance increased in more than 40% of the pathogen–antibiotic combinations monitored between 2018 and 2023 [1,2]. The discovery of new antimicrobial agents remains essential, yet therapeutic success depends on achieving appropriate drug exposure at the site of infection [3,4,5]. An antimicrobial may retain intrinsic activity while still failing clinically if local concentrations are insufficient or unevenly distributed at the target site [4,5]. Thus, the biological environment of infection can be as important as the intrinsic activity of the antimicrobial itself [3,6].

Biofilm-associated infections illustrate this challenge clearly. Within the structured extracellular matrix of a biofilm, gradients in oxygen, nutrients, metabolic products, and signaling molecules generate distinct local conditions over short distances [7]. Consequently, microbial populations within the same biofilm can differ in growth, metabolism, stress responses, and antimicrobial susceptibility [7,8]. Biofilm-associated tolerance is therefore multifactorial and should not be considered synonymous with genetically encoded resistance [8,9]. Biofilm-associated infections occur in diverse clinical settings, including oral and dental infections, chronic wounds, and implant- or device-associated infections, each presenting distinct barriers to effective treatment [10,11].

Restricted antimicrobial transport is only one component of this problem. Interactions with the biofilm matrix can influence local drug movement, while physiological differences among microbial subpopulations can alter their responses to the same antimicrobial exposure [8,12]. As a result, concentrations measured in blood or bulk tissue may not represent the exposure experienced throughout a structured infection. Physical access to the infection site alone does not guarantee biologically effective antimicrobial activity.

The challenge is temporal as well as spatial. Microbial physiology and local infection conditions can change during biofilm development and maturation. Conventional formulations commonly release drugs according to predetermined kinetics that may not reflect these changing conditions. The therapeutic challenge is not limited to delivering an antimicrobial to infected tissue. Drug exposure must match the location and biological state of the microbial population requiring treatment [7,8,13].

Conventional management of biofilm-associated wounds combines appropriate local wound care with treatment of clinically significant infection. Cleansing and debridement are used to remove devitalized tissue and reduce biofilm burden, while dressings support wound protection and management of the local wound environment. Systemic antimicrobial therapy is used when clinically indicated and is combined with appropriate source control and wound care. These measures remain essential, yet persistent biofilms can reform and reduce treatment effectiveness. Microorganisms within the wound may experience heterogeneous local conditions and reduced antimicrobial susceptibility [14,15]. The main components of conventional wound management, their key limitations, and the rationale for advanced antimicrobial delivery strategies are summarized in Figure 1.

Current strategies include localized delivery, targeted systems, controlled-release formulations, and systems activated by endogenous or external stimuli. Infection-associated changes in pH, redox conditions, and enzymatic activity may serve as triggers for antimicrobial release. External inputs can provide control over the timing of therapeutic activation [16,17,18]. Technological complexity alone does not guarantee therapeutic value. A nanoparticle (NP) is not inherently advantageous because of its size, and a responsive carrier is not useful simply because it detects a stimulus. The key question is whether the system overcomes a biological or pharmacological limitation that conventional treatment cannot adequately address [16,17].

This principle is particularly important in biofilm-associated infections and AMR, where genetically encoded resistance, restricted antimicrobial access, altered microbial physiology, and heterogeneous local conditions may coexist through distinct mechanisms [8,12]. A rational delivery strategy is best defined by the therapeutic limitation it aims to overcome, not by the identity or complexity of the carrier. This shifts attention from the delivery platform to its therapeutic function and provides the basis for considering spatial and temporal control as complementary design principles [16,18].

Spatial control concerns where an antimicrobial reaches and acts within an infected structure, whereas temporal control concerns when relevant exposure occurs and how long it is maintained. These dimensions provide the conceptual framework for spatiotemporally adaptive antimicrobial delivery [19,20]. Their translational value depends on selecting biologically relevant cues whose presence, magnitude, and variability are sufficiently characterized under relevant in vivo conditions. Infection-associated signals, including pH, redox conditions, enzymes, and other microenvironmental features, can vary across infection sites and biological contexts [21,22,23]. Clinical relevance further depends on evidence that this spatial or temporal control provides a measurable therapeutic advantage over appropriate conventional treatment [19,24].

Accordingly, this review examines antimicrobial drug delivery from a problem-oriented perspective. The discussion is organized around the biological and pharmacological barriers that limit treatment, including restricted access to infection sites, heterogeneous microbial states, biofilm-associated protection, and changes in the local infection environment. The review uses a spatiotemporal perspective to examine how antimicrobial exposure can be aligned with the location and changing biology of infection. Delivery systems are assessed according to the therapeutic function they provide, including improving local access, controlling exposure, responding to relevant biological signals, and supporting sequential treatment [21].

This perspective distinguishes the present review from conventional platform-based discussions of antimicrobial carriers. The emphasis is placed on the therapeutic problem first and the delivery technology second. A central question is whether additional formulation complexity produces a clear and measurable benefit compared with an appropriate conventional or simpler alternative. The following sections develop this perspective across spatial and temporal adaptation, infection-responsive delivery, biofilm-directed treatment, multimodal strategies, and the requirements for clinical translation (Figure 2).

Previous reviews have examined stimuli-responsive antimicrobial polymers, localized drug delivery for biofilm eradication, and stimulus-responsive nanosystems for bacterial infections [16,17,22]. The present review connects these areas by examining how antimicrobial delivery can be adapted to the spatial and temporal changes that occur during infection. It further considers sequential treatment, coordinated multimodal strategies, and the translational evidence needed to demonstrate a meaningful therapeutic advantage.

## 2. Literature Search Strategy

This narrative review was developed through a structured literature search to identify evidence relevant to antimicrobial drug delivery, infection-associated biological barriers, and strategies for improving antimicrobial exposure at the site of infection. Searches were conducted in PubMed, Scopus, Web of Science, and Google Scholar using combinations of terms related to antimicrobial drug delivery, controlled release, targeted delivery, biofilms, antimicrobial resistance, infection-responsive systems, stimuli-responsive delivery, external activation, local drug exposure, pharmacokinetics, pharmacodynamics, sequential delivery, and multimodal antimicrobial therapy. Search combinations were adjusted according to the main topic addressed in each section, including antimicrobial delivery, biofilm-associated barriers, responsive delivery, temporal control, and therapeutic translation. The final database search was conducted on 25 August 2026.

The search focused primarily on literature published between 2000 and 2026 to capture major developments in antimicrobial therapy, pharmaceutical technology, biofilm research, nanomedicine, and advanced drug delivery. Earlier landmark studies were retained when they provided foundational evidence on antimicrobial resistance, biofilm-associated infections, drug transport, controlled release, pharmacokinetic–pharmacodynamic relationships, or other concepts central to this review.

Eligible sources included peer-reviewed original research articles, systematic reviews, meta-analyses, narrative reviews, and authoritative reports published in English. Publications were retained when they were directly relevant to the predefined themes and provided mechanistic, comparative, therapeutic, or translational evidence relevant to the corresponding section. The main selection themes included barriers to antimicrobial action, infection-site drug exposure, spatiotemporal control of therapy, targeted and responsive delivery, controlled and sequential release, multimodal treatment, and the translational requirements of advanced antimicrobial delivery systems. Priority was given to studies providing mechanistic evidence, clinically relevant experimental data, comparative evaluations, or information relevant to therapeutic translation. Particular attention was given to primary studies with a defined delivery strategy, an appropriate comparator, a measurable antimicrobial or therapeutic outcome, or an infection-relevant experimental model. Reference lists of key articles and recent authoritative reviews were also screened to identify additional relevant publications.

Publications were excluded when they were not directly relevant to the objectives of the review, focused mainly on material properties without a clear link to antimicrobial delivery or therapeutic outcomes, lacked sufficient scientific detail, consisted of conference abstracts without an accompanying full-text publication, or duplicated previously reported findings. As this is a narrative review, formal systematic-review procedures, risk-of-bias assessments, and quantitative meta-analysis were not applied. Evidence from pharmaceutical sciences, microbiology, pharmacology, biomaterials, and drug delivery was critically synthesized to develop an integrated perspective on how antimicrobial exposure can be aligned with the location, biological state, and changing course of infection.

## 3. The Biological Mismatch Between Infection Dynamics and Conventional Drug Delivery

Conventional antimicrobial regimens are generally designed around predefined assumptions about dose, distribution, and duration of exposure. Infection biology, however, is neither spatially nor temporally uniform. Antimicrobial concentrations can vary across anatomical compartments and structured microbial communities, while host conditions and microbial physiological states change during infection. A regimen that appears pharmacologically adequate at one location or time point can produce different exposure elsewhere or later in the course of disease [25,26].

This mismatch occurs at several levels. Local barriers can restrict antimicrobial access, the infection microenvironment can change as disease progresses, and microbial populations can occupy physiological states with different susceptibilities. Uneven exposure can allow some subpopulations to survive because they experience insufficient or delayed antimicrobial activity. These factors can contribute to treatment failure even when the antimicrobial remains intrinsically active against the causative organism [26,27].

### 3.1. Heterogeneous Antimicrobial Exposure Within Infected Tissues

Effective antimicrobial therapy depends on drug exposure within the compartments occupied by microorganisms, not on adequate systemic concentrations alone. The route from the circulation to an infected site can involve vascular, interstitial, cellular, and microbial barriers. Because these barriers differ among tissues and disease states, plasma concentrations can differ from drug availability within infected compartments [28,29].

Biofilm-associated infection adds another level of spatial complexity. The extracellular matrix can influence antimicrobial transport through steric effects, binding, sequestration, and chemical interactions. These effects vary according to the antimicrobial and the composition and structure of the microbial community, creating local differences in drug availability [26,28].

Microbial architecture, matrix-rich regions, host-derived material, and anatomical confinement can further affect local diffusion. As a result, antimicrobial molecules can reach different regions at different concentrations and rates, while bulk measurements may not reflect exposure at the microscale [25,26]. A therapeutic concentration in blood or a tissue homogenate does not confirm that all microorganisms receive equivalent exposure. Some compartments may receive concentrations sufficient for effective killing, whereas others may experience lower or delayed exposure [28,29]. The central spatial challenge is clear: an administered dose does not automatically produce uniform antimicrobial exposure throughout a heterogeneous infection.

### 3.2. Temporal Evolution of the Infection Microenvironment

The biological environment of infection changes over time. Early microbial attachment can progress to aggregation and matrix production, followed by the development of structured communities with increasingly heterogeneous local conditions. The barriers encountered by an antimicrobial can consequently differ substantially between early and mature stages of infection [26,30].

Host responses introduce further temporal variation. Inflammation can alter vascular function, tissue permeability, immune activity, and local biochemical conditions. Persistent infection can sustain inflammatory signaling and contribute to tissue injury, while tissue damage can further modify the environment in which microorganisms and antimicrobials interact. In chronic wound infection, biofilm persistence, prolonged inflammation, and impaired tissue repair can coexist and reinforce one another [31,32,33].

Disease chronicity can further change therapeutic requirements. As infection persists, structured microbial communities, tissue damage, altered local physiology, and populations that have survived previous treatment can become increasingly important determinants of antimicrobial response [30,34]. An exposure profile established at the start of treatment cannot account automatically for biological changes that occur later. The key question is not simply whether an antimicrobial reaches the infected site, but whether its exposure remains appropriate as the local environment evolves.

### 3.3. Microbial Physiological Diversity and Antimicrobial Tolerance

Microbial populations within an infection are not physiologically uniform. Gradients in oxygen, nutrients, metabolic products, and other environmental factors can generate neighboring subpopulations with different metabolic activities and stress responses. These differences are particularly evident in biofilms, where cells separated by short distances can occupy distinct physiological states [7,8].

Physiological diversity has direct consequences for antimicrobial activity. Many antimicrobial agents act most effectively against microorganisms with particular growth or metabolic characteristics. Cells with reduced metabolic activity or altered physiological states can survive exposures that effectively eliminate more active populations. This contributes to biofilm-associated tolerance and remains distinct from genetically encoded resistance [9,12].

Dormant or transiently non-growing subpopulations can further complicate treatment. Persister cells are generally considered a phenotypically distinct subpopulation capable of surviving antimicrobial exposure without necessarily possessing heritable resistance. Their survival can contribute to post-treatment regrowth when antimicrobial concentrations decline and favorable growth conditions return [27,35].

Drug access and microbial susceptibility are therefore not equivalent. The same antimicrobial exposure can produce substantial killing in one physiological subpopulation while having limited activity against another. Treatment failure can arise from biological heterogeneity even when the antimicrobial reaches the infected structure and retains activity against the causative organism [26].

### 3.4. Pharmacological Selection Pressure and Resistance Emergence

Heterogeneous antimicrobial exposure can create distinct selective environments within an infected site. Where exposure is sufficient, susceptible cells may be eliminated, while microorganisms experiencing lower or transient concentrations can survive [28]. The relationship between antimicrobial exposure and resistance emergence is complex and cannot be defined by a single concentration threshold. Its consequences depend on the antimicrobial, microorganism, population size, treatment duration, and local biological conditions [26].

Incomplete killing can preserve surviving populations under antimicrobial pressure, while spatially heterogeneous infections can maintain microorganisms exposed to different pharmacological conditions. Biofilms are particularly relevant in this context because structured communities can contain physiologically diverse populations that remain viable during treatment. The emergence of heritable resistance through mutation or the acquisition and exchange of resistance determinants remains distinct from reversible phenotypic tolerance [26].

Repeated cycles of incomplete elimination are particularly relevant to chronic and recurrent infections. Surviving cells can repopulate the infected site after antimicrobial concentrations decline, and subsequent treatment can encounter a population that differs from the original population in physiological state, genetic composition, or both [27,35].

Static delivery strategies therefore face a challenge that extends beyond inadequate drug quantity. Effective therapy requires an appropriate relationship between antimicrobial exposure and a biological target that varies across space, changes over time, and contains physiologically diverse microbial populations. Table 1 summarizes the principal dynamic barriers that can disrupt this relationship and provides the biological basis for the adaptive design principles discussed in Section 4.

## 4. Spatiotemporal Adaptation as a Therapeutic Design Principle

In this review, spatiotemporal adaptation refers to the deliberate alignment of antimicrobial exposure with where treatment is needed, when it is needed, and how the biological conditions at the infection site change over time. The purpose is to address a defined limitation in antimicrobial treatment, such as restricted access, inadequate local exposure, or changes in microbial susceptibility.

### 4.1. Spatial Control: Defining Where Antimicrobial Exposure Is Required

Spatial control concerns the relationship between antimicrobial distribution and the location of the microbial population requiring treatment. The goal is not simply to increase drug accumulation within an infected organ or tissue. Effective treatment requires pharmacologically relevant exposure within the compartment containing the microbial target. Biofilms contain microscale chemical gradients and physiologically distinct subpopulations, creating local conditions that vary across an infected structure [7,36]. Bulk measurements of drug exposure may therefore fail to reflect the microscale environment experienced by microorganisms in regions with different oxygen availability, nutrient supply, or other determinants of physiology [13,36].

Restricted penetration does not explain every case of reduced biofilm susceptibility. In experimental *Pseudomonas aeruginosa* (*P. aeruginosa*) biofilms, tobramycin and ciprofloxacin penetrated the biofilm but failed to produce effective killing, showing that antimicrobial access alone does not determine treatment efficacy [37,38]. The value of spatial adaptation therefore depends on the biological limitation responsible for reduced activity. Improved distribution can be important when transport restricts access, while physiological heterogeneity can limit susceptibility even after an antimicrobial reaches the microbial population [38]. A rigorous spatial strategy requires identification of the microbial target, the barriers affecting access, and the biological conditions within the relevant compartment. The objective is to achieve effective exposure where antimicrobial activity is needed.

### 4.2. Temporal Control: Defining When Antimicrobial Exposure Is Required

Temporal control concerns the timing, rate, and duration of antimicrobial exposure. This dimension is important because microbial populations can occupy physiological states associated with different levels of metabolic activity and antimicrobial susceptibility [8]. Metabolic state can influence antibiotic efficacy and lethality [39,40]. Biofilm development can further change these conditions over time. In *P. aeruginosa*, oxygen limitation and nutrient availability contribute to changes in growth and antimicrobial tolerance as biofilms develop [38,41]. A fixed exposure profile can therefore encounter microbial populations with different physiological properties during treatment [13,39].

Temporal control is not simply prolonged drug release. A longer exposure period offers little advantage when the resulting concentration-time profile does not match the pharmacological requirements of the antimicrobial or the biological state of the target population [8,39]. This issue is particularly relevant to persister cells, which are phenotypic variants that can survive otherwise lethal antimicrobial exposure without stable genetic resistance and later resume growth [42,43]. Their abundance and survival can vary with growth conditions, nutrient availability, and treatment history [44,45]. Temporal adaptation therefore requires consideration of how antimicrobial requirements change during treatment. Depending on the biological context, therapy may require rapid drug availability, sustained effective exposure, or a planned change in the exposure profile. The appropriate pattern must be guided by a specific pharmacological need, not by formulation duration alone [13,39].

### 4.3. Integrating Spatial and Temporal Control

Spatial and temporal determinants of antimicrobial activity can interact within structured infections. In *P. aeruginosa* biofilms, simulated human exposure profiles of meropenem and tobramycin produced antibiotic-specific patterns of killing that varied across biofilm regions and changed with biofilm maturity [46]. Biofilm development can link spatial organization with changes in microbial physiology over time. In *Escherichia coli* biofilms, reduced energy metabolism and membrane potential accompanied biofilm growth, while metabolic exchanges between interior and peripheral populations contributed to survival and antibiotic tolerance [47].

Spatial variation can further influence bacterial responses during antimicrobial exposure. Microfluidic studies have shown that bacterial responses to antibiotic concentration gradients vary according to both location and time, revealing distinct concentration-dependent patterns [48,49]. The integration of spatial and temporal control is fundamentally a problem of exposure alignment. Spatial control determines whether an antimicrobial reaches the relevant microbial compartment. Temporal control determines how that exposure changes during treatment. Their combined importance increases when the location, physiological state, or treatment response of the target population changes over time. The goal is not maximum accumulation or prolonged exposure alone. It is an exposure pattern that matches the biological conditions governing treatment response.

### 4.4. Biological Relevance as the Basis of Adaptation

Adaptation has therapeutic value only when the factor used to modify antimicrobial exposure is relevant to the infection and the intended treatment objective. Infected tissues and biofilms can differ from surrounding environments through changes in pH, redox conditions, oxygen availability, microbial enzymatic activity, metabolites, and inflammatory processes [16,50,51]. The presence of such a feature alone does not establish its suitability for therapeutic control. A useful signal must occur within the relevant compartment and have sufficient magnitude, persistence, and reproducibility to support the intended change in antimicrobial exposure. Its distribution can vary according to the pathogen, anatomical site, disease stage, and local physiology [50].

Specificity is equally important. Some features associated with infection can occur during noninfectious inflammation or tissue injury. A biologically meaningful signal must therefore be evaluated according to its ability to distinguish the intended pathological environment, not simply its detectability [21,52]. The significance of conditions such as oxygen availability, pH, enzymatic activity, and metabolic state is highly context-dependent. A cue that performs reliably in one infection model may be weak, absent, or heterogeneous in another [51,53].

An adaptive response is meaningful only when a change in the selected biological feature corresponds to a genuine therapeutic requirement. Otherwise, stimulus detection can alter drug release without improving treatment response [22,50]. A biologically informed design requires a clear causal rationale linking the selected signal to the intended therapeutic function. Three questions are central: Does the signal identify the relevant infection compartment? Is its magnitude sufficient to produce a reliable response? Does the resulting change in exposure address a defined pharmacological limitation [16,50,54]?

Spatiotemporal adaptation should be distinguished from several established delivery approaches. Controlled release primarily regulates the rate or duration of drug release and does not necessarily require a response to a changing biological signal [16,17]. Stimuli-responsive or triggered delivery uses a defined biological or externally applied signal to alter drug release, carrier behavior, or therapeutic activity [18,19,20,21,22,23]. Such a response can provide one component of a spatiotemporal strategy, but the presence of a trigger alone does not make a system spatiotemporally adaptive. Targeted delivery focuses mainly on where a drug is distributed or retained. In antimicrobial delivery, targeting may involve passive localization or active recognition of a specific microbial or tissue-associated feature [20,23]. Targeting can improve spatial delivery, but spatial targeting alone does not establish temporal adaptation. Sequential treatment refers to administering interventions in a planned order when the effect of one intervention can influence the activity of the next. Sequential treatment can therefore contribute to spatiotemporal adaptation, but it is not synonymous with it.

Spatiotemporal adaptation is therefore a broader therapeutic framework rather than a single delivery technology. It asks whether antimicrobial distribution and exposure over time are matched to the biological state and treatment needs of the infection. Controlled release, stimuli-responsive or triggered delivery, targeted delivery, and sequential treatment can each contribute to this framework, but none of them alone is equivalent to spatiotemporal adaptation. Here, “adaptive” is used in a functional sense. It does not imply that the delivery system independently senses, decides, and adjusts its treatment. Instead, a system is considered adaptive when its design links antimicrobial distribution or exposure to a relevant biological condition or stage of treatment.

Biological relevance provides an appropriate basis for judging technological complexity. Additional sensing or control mechanisms are justified only when they produce a measurable advantage over a simpler alternative [50]. The next question is how this adaptation can be initiated. Section 5 examines the biological and externally applied signals that can govern therapeutic activation.

## 5. Biological and External Triggers for Adaptive Therapeutic Activation

Once the therapeutic need for spatial or temporal adaptation has been defined, the next question is how the response should be initiated. Adaptive antimicrobial activation requires a signal that can be translated into a defined therapeutic event. Such signals can arise from the infected microenvironment or be applied externally as controlled physical inputs. Endogenous triggers reflect local biological conditions, whereas external triggers allow activation at a selected time [19,55]. This section focuses on the signals that initiate activation and the mechanisms through which they act, independent of the carrier used to detect or respond to them.

### 5.1. Endogenous Infection-Associated Triggers

Endogenous triggers arise from biochemical and physiological features of the infected microenvironment. Their main advantage is the potential to link therapeutic activation to local conditions without external stimulation. Their usefulness depends on the biological specificity, spatial distribution, and consistency of the signal across the infection settings in which activation is intended [56].

#### 5.1.1. Chemical, Redox, and Metabolic Cues

Local pH can serve as an activation signal because microbial metabolism and infection-associated processes can alter acidity within infected regions. pH-responsive systems generally rely on protonation or acid-sensitive chemical transformations that occur within a defined pH range. The usefulness of pH as a trigger remains site dependent because local acidity can vary across anatomical sites and within heterogeneous microbial communities [16,53,56].

Redox-responsive systems use local oxidative or reducing conditions to induce a chemical or physicochemical change. Reactive oxygen species are relevant because infection and inflammation can alter the local redox environment. Elevated oxidative activity, however, is not specific to bacterial infection [17,56]. Reduced oxygen availability provides another endogenous cue. Hypoxic conditions can occur in poorly oxygenated infected tissues and deeper biofilm regions and can support conditional activation [23,53].

Metabolic signals can provide information about local biological activity. Bacterial metabolites can serve as triggers when their presence or concentration can be linked to a defined and reproducible activation mechanism [56]. These signals represent different biological features: pH reflects local chemical conditions, redox signals reflect the oxidative environment, hypoxia reflects oxygen availability, and metabolites reflect microbial or local metabolic activity [57,58].

A practical example is a pH-sensitive hydrogel developed for drug-resistant biofilm-infected diabetic wounds. Fan et al. combined an antimicrobial peptide with a photosensitizer in a hydrogel designed to release its therapeutic components under acidic conditions. The system showed antibacterial activity and supported wound healing in experimental models, illustrating how local pH changes can guide antimicrobial delivery [59].

#### 5.1.2. Enzymatic and Microbial Signals

Enzymes represent a distinct class of endogenous triggers because they can directly catalyze cleavage, degradation, or structural changes in a responsive component. Their usefulness depends on sufficient enzymatic activity at the target site and on the ability of the system to distinguish the relevant pathological environment from surrounding tissues [16,56].

Microbial products, including secreted enzymes, metabolites, toxins, and signaling molecules, provide additional endogenous signals. Because these cues arise directly from microbial activity, they can offer a closer connection to the targeted biological process than general host-associated changes. Their usefulness depends on how consistently they are produced across the microorganisms and infection states of interest [18].

The main limitation of endogenous triggering is biological variability. A signal can be present in one region of an infection and weak or absent in another. Its magnitude can change with pathogen composition, disease stage, host response, and local physiology. Endogenous activation is most convincing when the relationship between the selected signal and the intended activation event has been demonstrated in the specific biological setting of use [56].

### 5.2. Externally Controlled Triggers

Externally controlled activation uses a physical input that is applied independently of the local biological state. Light, magnetic fields, temperature, electrical stimulation, and ultrasound have been investigated as means of controlling antimicrobial activation. Their main advantage is direct control over the timing of stimulation. Their practical value depends on whether the applied energy can reach the target site and produce the intended therapeutic response [18].

Light can initiate photochemical reactions or generate local heating through photothermal conversion. Optical activation can provide precise temporal control, although tissue penetration depends strongly on wavelength and target depth [16,18].

Magnetic-field activation can enable remote control when a magnetic field interacts with a system capable of converting that input into a therapeutic response. Depending on the underlying mechanism, magnetic fields have been used for functions such as magnetic hyperthermia and field-directed localization [16]. Thermal activation relies on temperature-dependent changes in a responsive system. Heating can be applied directly or generated through externally delivered energy, including photothermal or magnetic heating [16,18].

Electrical stimulation can induce electrochemical or electrically driven physicochemical changes in responsive therapeutic systems. Its usefulness depends on the mechanism of energy transfer and the practical feasibility of delivering electrical stimulation to the treatment site [18]. Ultrasound provides an acoustic input that can reach deeper tissues than optical irradiation. Depending on the responsive system and acoustic conditions, ultrasound can generate mechanical effects, cavitation, localized heating, or phase transitions that alter antimicrobial release or local transport [60].

A practical example was reported by Zong et al., who developed an ultrasound-responsive hydrogel containing peptide NPs for diabetic wounds with biofilm infection. Ultrasound promoted hydrogel disruption and cargo release, while the resulting smaller fragments showed deeper penetration into the wound. This example illustrates how external stimulation can control local antimicrobial delivery and improve access to the treatment site [61].

External triggers differ primarily in the type of energy applied and the mechanism through which that energy produces activation. No single modality is universally advantageous. Selection depends on target depth, anatomical accessibility, the required degree of temporal control, and compatibility between the stimulus and the intended activation mechanism [16,18].

### 5.3. Multi-Trigger Strategies

Multi-trigger strategies combine more than one activating signal within a single therapeutic design. The inputs can include two endogenous cues, two externally applied stimuli, or a combination of endogenous and external signals. These approaches can incorporate multiple sources of biological or physical information when a single signal lacks sufficient specificity or consistency across a heterogeneous infection [16,18].

One approach uses combined responsiveness, in which multiple stimuli contribute to the activation event. Examples include pH- and enzyme-responsive systems, pH- and temperature-responsive systems, and combinations involving pH with reactive oxygen species or photothermal stimulation [16]. The potential advantage is greater conditional control because activation can depend on multiple relevant inputs instead of a single cue.

A second approach uses sequential activation, in which different signals control distinct stages of the therapeutic process. This principle has been demonstrated in a biofilm system in which a magnetic field guided an antibiotic-containing system toward *P. aeruginosa* biofilms, followed by ultrasound-induced phase transition and cavitation that promoted biofilm disruption and antibiotic penetration [60]. The critical feature of this strategy is the functional relationship between the signals and the stages of treatment.

Multi-trigger strategies are justified when each additional signal provides distinct therapeutic information or enables a separate stage of control. Adding multiple inputs without a clear mechanistic purpose increases system complexity without necessarily improving selectivity or therapeutic performance. Their value should be judged by the additional conditionality, precision, or functional sequencing they provide [16,18]. The principal endogenous and externally applied triggers are summarized in Table 2 according to their signal source, activation mechanism, potential therapeutic value, and major limitations.

## 6. Navigating Biofilm Architecture Through Sequential Therapeutic Action

Biofilm-directed therapy may require more than the concurrent use of agents with different activities. Established biofilms contain structural barriers, protected microbial niches, local conditions that promote tolerance, and physiologically distinct surviving populations. Sequential treatment becomes relevant when one intervention must first modify a barrier or biological condition that limits the effectiveness of the next [64,65].

The sequence described here is conceptual, not universal. Depending on the biofilm, therapeutic mechanism, and infection context, individual actions may overlap or occur simultaneously. The central principle is that treatment should account for dependencies among biological barriers instead of assuming that a single intervention can overcome every component of biofilm-associated protection [66,67].

### 6.1. Crossing the Extracellular Polymeric Substance (EPS) Matrix

The first therapeutic requirement is to gain access beyond the outer regions of the biofilm and maintain access to its interior. The biofilm matrix, composed largely of EPS, is a dynamic environment whose components can influence the movement, retention, and local availability of antimicrobial agents. The objective is not simply to enter the biofilm, but to achieve effective exposure within deeper microbial compartments [65].

Matrix-directed interventions can facilitate this process when specific matrix components restrict access. Experimental studies have shown that degradation of extracellular DNA or other matrix constituents can improve subsequent antimicrobial activity, although the magnitude of this effect depends on the composition of the particular biofilm [66,68].

Crossing the matrix is not equivalent to eradicating the biofilm. Its therapeutic purpose is to reduce a structural barrier that limits subsequent antimicrobial action. Success at this stage should be judged by whether matrix modification improves access or enables a later therapeutic step, not by structural disruption alone [65,69].

### 6.2. Accessing Heterogeneous Microbial Niches

Improved access to the biofilm does not ensure equal exposure of all resident microorganisms. The challenge at this stage is to extend effective antimicrobial action from readily accessible regions to microbial populations located in compartments that remain less reachable or less affected [64].

This distinction becomes especially important after partial treatment. Surviving cells may occupy regions that were incompletely reached during the preceding step. Their persistence does not necessarily indicate that the antimicrobial itself lacks intrinsic activity. In mature *P. aeruginosa* biofilms, reducing matrix-associated sequestration of tobramycin improved its diffusion beyond the biofilm periphery, illustrating how changes in intrabiofilm distribution can improve access to protected regions [70].

The goal of this stage is not simply to increase the overall antimicrobial concentration. It is to progress from initial biofilm entry to effective exposure of microbial compartments that remain protected after the preceding intervention.

### 6.3. Remodeling or Disrupting the Biofilm Microenvironment

Once access has been established, further treatment may require modification of conditions that continue to support the protected biofilm state. Enzymatic strategies can alter extracellular structural components without directly killing bacteria, thereby changing the environment in which subsequent antimicrobial activity occurs [66,71].

This principle has been demonstrated experimentally. A tri-enzymatic cocktail dispersed mature biofilms formed by several bacterial species, and subsequent antibiotic treatment produced lower residual viable counts than either intervention alone. These findings support the concept that structural modification can create conditions more favorable for a subsequent antimicrobial step [66].

Biofilm remodeling can involve interference with regulatory processes that support collective microbial behavior. Quorum-sensing inhibition has increased antibiotic susceptibility in experimental biofilms, although the magnitude and direction of this effect depend on the organism, antimicrobial agent, and model system. Its therapeutic value is therefore context-dependent [72,73,74].

The purpose of this stage is not disruption for its own sake. The objective is to modify a structural or biological condition that supports microbial survival and create a more favorable state for the next therapeutic action. Matrix disruption must be coupled with effective antimicrobial killing because dispersal alone does not establish elimination of released or residual microorganisms [75].

### 6.4. Eliminating Residual Tolerant Populations

After structural and local biological barriers have been modified, the surviving population may differ substantially from the population present at the beginning of treatment. Residual cells can include microorganisms whose physiological state confers pronounced antimicrobial tolerance, including persisters and other metabolically distinct subpopulations. Their survival should be addressed according to the mechanism responsible for persistence instead of simply repeating the preceding intervention [76].

Persisters are particularly relevant because they can survive otherwise lethal antimicrobial exposure without possessing stable genetic resistance. More broadly, residual populations may include slow-growing or metabolically altered cells whose physiological state reduces susceptibility to antimicrobial mechanisms that depend on active cellular processes [76,77].

In a catheter-associated *Staphylococcus aureus* biofilm model, cells with characteristics associated with persistence showed increased survival during antimicrobial challenge. Their tolerance remained after enzymatic matrix disruption, demonstrating that removal of a structural barrier does not necessarily eliminate a physiological barrier [76].

Metabolic manipulation provides one example of how this final obstacle can differ from the initial biofilm barrier. Allison et al. demonstrated that selected metabolites could restore aminoglycoside killing of persister cells by promoting metabolic activity and drug uptake. These findings show that eliminating surviving populations may require restoration of antimicrobial susceptibility, not simply greater drug exposure [78].

The final therapeutic objective is to address the biological basis of residual survival. Sequential treatment should ultimately aim to eliminate populations capable of sustaining persistence or driving subsequent regrowth, not merely reduce biofilm biomass [77,78].

### 6.5. Why Sequence Matters

The value of sequential treatment lies in therapeutic dependency. A matrix-directed intervention may improve access to protected regions, modification of local conditions may enhance subsequent antimicrobial activity, and residual populations may require a distinct strategy based on the mechanism responsible for their survival [66,68]. The rationale for sequencing is not that every biofilm requires the same order of interventions. Instead, the effectiveness of a later action may depend on first modifying a limitation that prevents it from working effectively [67].

The therapeutic sequence should be determined by the dominant limitations of the specific biofilm and the relationship between successive interventions. Limited access, restricted penetration, unfavorable local conditions, reduced antimicrobial susceptibility, and residual survival represent distinct therapeutic problems. Each may require a different intervention [64,77]. Figure 3 summarizes how spatiotemporal adaptation can support localized and time-controlled antimicrobial exposure.

## 7. Therapeutic Architectures for Implementing Spatiotemporal Adaptation

The therapeutic principles described above must be implemented through an appropriate physical architecture. These systems should be compared according to the functions they provide, including localization, access, responsiveness, temporal control, and persistence, not simply according to material novelty [79,80]. No architecture is intrinsically superior across all infection settings. The appropriate system depends on the therapeutic requirement and the biological and pharmacological constraints of the intended application. Material selection should follow the function required for treatment.

The implementation of spatiotemporal antimicrobial delivery is not limited to nanoscale carriers. Localized depots and hydrogels, surface-associated formulations, and biomimetic or biological systems can provide sustained exposure, site-specific retention, controlled release, or biologically derived therapeutic functions [17,81,82]. These approaches should be selected according to the infection setting and therapeutic need, not technological scale. The following sections compare nanoscale and non-nanoscale architectures according to the functions they provide and the factors that influence their clinical development.

### 7.1. Nanoscale and Hybrid Carrier Systems

Nanoscale carriers provide a versatile approach to modifying antimicrobial delivery through control of drug encapsulation, carrier structure, surface properties, and release behavior. Polymeric, lipid-based, inorganic, and hybrid NPs have been investigated for improving antimicrobial performance against biofilm-associated and other structured infections [79,83].

Polymeric NPs can provide structural stability, chemical functionalization, and tunable release characteristics. Their value, however, depends on whether these properties improve antimicrobial performance within the relevant infection environment [84]. Lipid-based systems offer different structural and interfacial properties and have been explored for antibiotic delivery to biofilms [83]. Recent work on aaptamine-loaded fusogenic liposomes reported sustained release and enhanced antibacterial activity against methicillin-resistant *Staphylococcus aureus*, illustrating how membrane fusion can improve antimicrobial delivery [85].

Inorganic NPs may contribute functions beyond drug carriage, including intrinsic antimicrobial activity and other therapeutic effects [79,80]. Their value should be assessed according to the specific contribution of the inorganic component. Hybrid nanosystems combine components with complementary properties. Lipid–polymer, organic–inorganic, and membrane-coated systems are examples in which different structural elements contribute distinct functions [86]. A lipid–polymer hybrid NP developed for localized and sustained antibiotic delivery against bacterial biofilms illustrates how hybridization can modify antimicrobial exposure without adding complexity solely for its own sake [87].

Nanoscale dimensions alone do not ensure effective access throughout an infected structure. Carrier movement depends on particle properties and interactions with the surrounding biological environment, making direct investigation of NP behavior within biofilms essential [83]. The key translational question is whether nanoscale design provides a measurable advantage over the free antimicrobial or a simpler formulation while maintaining acceptable safety and manufacturing reproducibility [79,88].

### 7.2. Localized, Depot, and Surface-Associated Systems

Localized architectures are designed primarily to retain therapeutic material at or near an accessible infection site. Hydrogels, injectable depots, wound systems, and surface-associated formulations can provide sustained local antimicrobial availability while limiting prolonged systemic exposure [17,89].

Adhesive and bioadhesive systems can improve local retention by maintaining close contact between the formulation and the wound or tissue surface, thereby supporting localized antimicrobial delivery [90]. Fibrous and nonwoven architectures, particularly electrospun nanofiber systems, provide porous matrices that can incorporate antimicrobial agents, support controlled local release, and function as wound-covering materials [91,92].

Ribbon-like nanostructured materials, including bacterial cellulose networks composed of ultrafine fibrils, represent another wound-dressing architecture. Their high-water retention and large surface area can support functionalization with antimicrobial components [93]. The value of these architectures depends on whether their structural properties improve therapeutic retention, drug release, or antimicrobial activity in the intended infection setting.

Hydrogels are water-rich polymeric networks whose composition and structure can be adapted for controlled local delivery and the incorporation of additional bioactive functions. Depending on their design, these systems can provide sustained release and other functions relevant to treatment [17].

For biofilm-associated infections, a major advantage of hydrogels is local persistence. They have been investigated as delivery matrices for antibiotics, antimicrobial peptides, enzymes, and bacteriophages, including systems responsive to local pH, enzymatic activity, or reactive oxygen species [17]. In wound applications, hydrogels can provide both a local therapeutic matrix and a dressing environment. Their activity against biofilm-associated microorganisms, however, should be demonstrated directly and not inferred from planktonic assays [94,95].

Surface-associated systems operate over a more restricted spatial range. Antibacterial hydrogel coatings have been developed to modify implant surfaces and interfere with biofilm formation at biomaterial interfaces [81,96]. Their principal advantage is site-specific activity, although their therapeutic influence remains concentrated near the treated surface.

Local persistence and access to the microbial target should be considered separately. A depot may retain a therapeutic agent at the treatment site while diffusion beyond the matrix remains insufficient to reach deeper infected compartments [17]. This distinction is important when selecting localized systems and determining whether additional strategies are needed to improve transport. Translation of advanced local systems requires evidence that their responsive or multifunctional behavior remains effective under biologically relevant conditions. Current analyses emphasize the need for predictive biofilm models, mechanistic validation, and clinically meaningful evaluation before increasingly complex hydrogel systems can be confidently advanced [17,97].

### 7.3. Biomimetic and Biological Therapeutic Systems

Biomimetic and biological architectures incorporate functions derived from biological structures or agents. This category is mechanistically diverse. Some systems primarily modify delivery behavior, whereas others possess intrinsic antimicrobial activity [82,98].

Cell membrane-coated NPs combine an engineered core with naturally derived membrane material. In bacterial infection research, they have been investigated for antimicrobial delivery, infection targeting, toxin neutralization, and combination therapy [82,99]. Their performance depends on the membrane source and the preservation of relevant biological properties. Clinical development further requires reproducible production, adequate stability, and acceptable immune compatibility [88].

Extracellular vesicles provide another biologically derived nanoscale architecture. Their ability to transport proteins, lipids, and nucleic acids has stimulated interest in vesicle-based therapeutic and antimicrobial delivery platforms [100,101]. Their biological origin introduces challenges related to heterogeneity, immunological interactions, manufacturing, and regulatory standardization [102,103].

Bacteriophages should be distinguished from biological delivery vehicles because phages can function as antibacterial agents themselves. Formulations such as hydrogels, liposomes, and responsive polymers can modify phage stability, retention, or release [104,105]. In these systems, the delivery architecture supports phage activity rather than producing the primary antibacterial effect.

The value of biological architectures lies in the specific function contributed by their biological components. Their use is most justified when biological recognition, interaction, or intrinsic activity provides an advantage that a simpler synthetic design cannot adequately achieve.

### 7.4. Multifunctional and Hierarchical Therapeutic Architectures

Multifunctional architectures integrate more than one delivery or therapeutic function within a coordinated system. Their rationale is strongest when the therapeutic objective cannot be achieved by a single functional component [17,86]. How these functions are integrated is equally important. Some systems combine functions that operate concurrently, such as local retention and controlled release. In other architectures, one function creates conditions required for the next action, producing a hierarchical relationship between components [86,87].

This distinction should guide the evaluation of complex systems. The presence of multiple functional elements does not itself demonstrate therapeutic benefit. A multifunctional architecture should establish the contribution of each component and demonstrate an advantage over an appropriate simpler comparator [17,86]. Greater integration increases the demands for characterization, stability assessment, manufacturing control, and biological evaluation [17,88,103]. The goal is not maximal functionality, but the minimum complexity required to achieve the intended therapeutic effect.

### 7.5. Comparative Perspective

No single therapeutic architecture can optimize every dimension of antimicrobial delivery. Nanoscale carriers can modify drug transport, protection, and release, whereas localized and depot-based systems are particularly suited to maintaining therapeutic material at a defined site [17,83].

Surface-associated systems provide persistent activity at a treated interface but have limited spatial reach. Biomimetic and biological architectures may offer distinctive biological interactions or intrinsic antimicrobial activity, although they present greater challenges related to heterogeneity and standardization [96,103]. Multifunctional systems can address several therapeutic requirements within one design. Increasing integration, however, adds translational complexity and requires clear evidence of functional benefit [17,86].

The choice of architecture should begin with the treatment problem, not the available material. Once the required degree of localization, access, responsiveness, temporal control, and persistence has been defined, material composition can be selected accordingly. Table 3 compares the major therapeutic architectures according to their potential for localization, access, responsiveness, temporal control, and persistence, together with the principal limitations affecting translation. These capabilities represent architecture-level potential, not universal performance rankings.

The clinical value of these architectures cannot be judged by technological sophistication alone. Localized systems may offer a practical advantage when prolonged exposure is needed at an accessible infection site, whereas nanoscale or biological systems may be more relevant when transport barriers or biological recognition limit treatment. Multifunctional designs may be justified for complex infections, although each additional component increases the demands for characterization, manufacturing, and regulatory evaluation. Future development should focus on identifying the simplest architecture that can provide a measurable therapeutic advantage in the intended clinical setting [106].

## 8. Coordinated Multimodal Antimicrobial Therapy

Spatiotemporal control can improve where and when antimicrobial exposure occurs. Complex infections, however, may involve several limitations that cannot be addressed by a single therapeutic mechanism. Multimodal therapy is justified when different interventions provide complementary functions. Its value depends not on the number of components, but on whether their activities are mechanistically compatible and therapeutically coordinated. One intervention may increase susceptibility to another, act through an independent mechanism, or target populations that survive the primary treatment. In some combinations, one component can interfere with the biological requirements of another, leading to reduced activity or antagonism [107,108].

### 8.1. Antibiotic-Based Combination Strategies

Antibiotic combinations should be selected on the basis of complementary pharmacological properties and a clear therapeutic rationale. Depending on the agents involved, a combination can broaden effective activity, enhance bacterial killing, or reduce the likelihood that a subpopulation escapes a single mechanism. These effects are not universal. The outcome can vary according to the drug pair, bacterial isolate, exposure conditions, and method used to evaluate the interaction [107].

The timing of antibiotic exposure can influence treatment activity. Simultaneous administration is appropriate when concurrent exposure is compatible with the pharmacology of both agents. Sequential administration may be more effective when the first treatment changes a property that influences the activity of the second. Treatment order should be tested experimentally and not treated as an arbitrary feature of the regimen.

The performance of an antibiotic combination should not be judged solely by comparison with either monotherapy. Its combined effect should be evaluated against the activity expected from the individual agents under comparable conditions. This distinction is important because interactions can range from antagonism to synergy and may vary with concentration and exposure time [107,109].

### 8.2. Complementary Non-Antibiotic Antimicrobial Modalities

Non-antibiotic agents can address therapeutic limitations that increasing antibiotic exposure alone cannot resolve. Antimicrobial peptides, bacteriophages, antimicrobial enzymes, nucleic-acid-based interventions, and materials with direct antimicrobial activity have distinct mechanisms and therapeutic roles. Each approach should be evaluated according to its specific biological function [110,111].

A recent biofilm-directed example illustrates how complementary antimicrobial functions can be integrated within a single treatment system. Li et al. developed a biofilm microenvironment-responsive platform that combined gas therapy, antibacterial metal ions, photothermal therapy, and chemodynamic therapy. The integrated design targeted biofilm-associated barriers and bacterial survival through multiple mechanisms. This example shows how a multimodal system can address several relevant therapeutic limitations when those mechanisms serve complementary functions [112].

Antimicrobial peptides often act through mechanisms that differ from those of conventional antibiotics, including interactions with bacterial membranes. This mechanistic difference can provide useful complementarity in selected combinations. Some peptide–antibiotic pairs have shown enhanced antimicrobial or antibiofilm activity. The outcome, however, depends on the agents, microorganism, and exposure conditions [113].

Bacteriophages provide another form of antibacterial activity through their interaction with specific bacterial hosts. Selected phage–antibiotic combinations have shown enhanced killing and antibiofilm activity, including against multidrug-resistant organisms. These effects are not universal, and antagonism can occur. Bacterial susceptibility to the phage, antibiotic mechanism, phage replication, and treatment timing should all be considered when designing these combinations [114,115].

Antimicrobial enzymes add further mechanistic diversity by acting on defined microbial or biofilm-associated substrates. Phage-derived endolysins, for example, degrade bacterial cell-wall structures. Experimental combinations of endolysins and antimicrobial peptides have shown enhanced antibiofilm activity. These findings demonstrate how agents directed at different biological targets can complement one another [116].

Nucleic-acid-based approaches differ from direct antimicrobial therapies because they can modulate selected microbial genes or pathways. Their therapeutic value depends on the relevance of the chosen target and on whether its modulation improves treatment response. Intracellular delivery and target accessibility remain major challenges. These approaches should be evaluated according to the biological relevance of their targets and their ability to improve therapeutic outcomes [117,118].

### 8.3. Energy-Activated Antimicrobial Modalities

Energy-activated modalities should be distinguished from the externally controlled triggers discussed in Section 5.2. In these approaches, light or ultrasound contributes directly to antimicrobial activity instead of simply initiating drug release. Photodynamic therapy combines a photosensitizer, light, and molecular oxygen to generate reactive species that damage microbial cells and biofilm-associated structures. This mechanism differs from conventional antibiotic action and can complement antibiotic treatment. Its performance depends on the photosensitizer, light delivery, oxygen availability, and local infection environment [119].

Photothermal therapy converts absorbed light into localized heat. The resulting thermal stress can damage microbial structures and alter local conditions in ways that support chemical antimicrobial activity. Photothermal and photodynamic approaches can be combined when heat and oxidative damage serve distinct therapeutic functions. Their usefulness depends on delivering sufficient energy to the infected site while limiting injury to surrounding tissues [120,121].

Ma et al. reported a hydrogel for biofilm-infected chronic wounds that combined controlled nitric oxide delivery with photothermal activation. Sustained nitric oxide release supported biofilm disruption, while near-infrared irradiation produced localized heating and accelerated nitric oxide release. The study illustrates how externally applied energy can complement a chemical antimicrobial mechanism when both interventions provide distinct therapeutic benefits [122].

Sonodynamic therapy combines ultrasound with a sonosensitizing agent to generate localized cytotoxic effects, including reactive oxygen species. Ultrasound can facilitate energy delivery to less accessible sites. Its activity depends on acoustic parameters, sensitizer behavior, oxygen conditions, and biological heterogeneity. Its therapeutic value must therefore be judged according to the specific limitation it is intended to address [123].

The presence of several antimicrobial mechanisms does not, by itself, establish synergy. Physical and chemical modalities should be compared with their individual components under matched conditions to determine whether the combination provides an additional therapeutic benefit [119,124].

### 8.4. Therapeutic Coordination, Sequence, and Synergy

The central challenge in multimodal therapy is therapeutic coordination. Interventions can be administered simultaneously or sequentially, and these approaches are not necessarily equivalent. Simultaneous treatment may be appropriate when complementary activities remain compatible during concurrent exposure. Sequential treatment becomes particularly relevant when the first intervention creates conditions that improve the activity of the next [108,125].

Order-dependent effects can arise when one modality changes membrane permeability, microbial physiology, a protective structure, or another determinant of susceptibility. The optimal sequence cannot be predicted solely from the independent mechanisms of the individual components. The same combination may produce different outcomes when the interval between treatments changes. Sequence should consequently be established experimentally in the relevant biological setting [125,126]. Figure 4 illustrates the general distinction between simultaneous and sequential coordination without prescribing a universal treatment order.

The term synergy requires equally careful use. Greater activity than either monotherapy does not, by itself, demonstrate synergy. The combined effect must be compared with an appropriate expectation based on the activities of the individual components. Checkerboard assays examine antimicrobial interactions across different concentration combinations and commonly use the fractional inhibitory concentration index for interpretation. Time-kill studies measure changes in microbial viability over time and can reveal interactions that are not apparent from a single endpoint [107,125]. Quantitative models, including Bliss independence and Loewe additivity, compare the observed combination effect with an expected effect based on the activity of the individual treatments [109]. Different approaches can produce different conclusions because they rely on distinct experimental conditions and underlying assumptions. Synergy should be assessed using methods that match both the therapeutic combination and the biological question.

Multimodal therapy should be designed as a coordinated set of complementary therapeutic functions. Each component needs a defined role, a clear mechanistic relationship with the other interventions, and a timing strategy supported by experimental evidence. The central question is not how many therapeutic components are included. It is whether their coordinated use produces a reproducible advantage over an appropriate simpler regimen [106,107,109].

## 9. Translational Design and Evaluation of Advanced Antimicrobial Delivery Systems

The delivery strategies discussed in the preceding sections aim to improve antimicrobial therapy by controlling where, when, and for how long a drug acts. An advanced delivery function alone, however, does not establish therapeutic value. Improved loading, controlled release, stimulus responsiveness, or local retention matter only when they produce a measurable benefit in the intended treatment setting. This section examines how these benefits can be evaluated during the transition from formulation design to clinical development. Advanced antimicrobial delivery should be assessed through a clear connection between formulation design, local drug exposure, biological response, and therapeutic benefit [127,128].

Formulation complexity creates an additional translational burden. Each material or functional component can introduce new sources of variability, alter biological behavior, complicate manufacturing, or expand safety requirements. Complexity is justified only when each component addresses a defined therapeutic limitation and contributes to a measurable benefit. Evaluation should focus on the clinical purpose of a system, not simply on the number of functions it contains [129,130].

### 9.1. Linking Formulation Design to Therapeutic Performance

Formulation development should begin with a clearly defined therapeutic problem. The intended infection, route of administration, treatment objective, and clinical limitation should guide the required delivery profile. An acute infection requiring rapid microbial killing may need a different exposure pattern from a chronic, biofilm-associated, or device-related infection. No delivery architecture is inherently superior outside the biological and clinical context for which it was designed [127,128].

This distinction separates formulation properties from therapeutic outcomes. Particle size, surface characteristics, drug loading, structural stability, and release behavior describe the formulation itself. Their clinical importance depends on whether they improve the interaction between the antimicrobial agent, the infected site, and the target microbial population. A favorable physicochemical property is not, by itself, a therapeutic endpoint [131].

The same principle applies to sustained release, local retention, and stimulus responsiveness. Prolonged release is useful only when the resulting exposure profile supports the pharmacological requirements of the antimicrobial agent. Increased retention has value only when the retained drug remains active and well tolerated. Similarly, a responsive system is useful when activation produces a meaningful change in drug availability at the relevant site and time [50].

Evaluation should follow a clear sequence: formulation characteristics influence drug release and transport, which determine local exposure, biological activity, and ultimately therapeutic benefit. Each step requires direct evidence. Antimicrobial activity alone does not demonstrate improved delivery, and the presence of a targeting or responsive component does not prove that it contributes to treatment. The proposed delivery mechanism should be supported by evidence linking key formulation attributes to the observed biological effect in the relevant infection setting [128,131].

Representative primary studies in this review illustrate the importance of linking delivery design to the model, comparator, and therapeutic endpoint. Baek et al. evaluated a lipid–polymer hybrid nanoparticle for localized and sustained antibiotic delivery against Gram-positive and Gram-negative bacterial biofilms. The study compared the hybrid system with free antibiotics and non-hybrid formulations and reported substantially improved biofilm inhibition at lower antibiotic concentrations [87]. The findings support the value of modifying local antimicrobial exposure, but the evidence was generated primarily in vitro and does not establish whether the observed advantage would persist in infected tissues or translate into improved clinical outcomes.

Ma et al. provided stronger infection-relevant evidence by evaluating a photo-modulated nitric oxide-releasing hydrogel in biofilm-infected chronic wounds, including an MRSA-infected diabetic mouse wound model. Treatment effects were assessed using bacterial burden and wound-healing outcomes, including tissue remodeling, inflammatory responses, angiogenesis, and collagen deposition [122]. The study demonstrates how controlled local delivery combined with external activation can produce both antimicrobial and tissue-level benefits. However, the need for external near-infrared irradiation and the reliance on a single small-animal wound model remain important translational limitations.

Geng et al. reported another example using aaptamine-loaded fusogenic liposomes against MRSA. Compared with free aaptamine, the liposomal formulation showed sustained release and enhanced antibacterial activity [85]. This study supports the potential of carrier design to improve antimicrobial performance, but its evidence remains primarily at the formulation and in vitro antibacterial level rather than demonstrating therapeutic benefit in an infection-relevant animal model.

These studies illustrate different levels of evidence rather than a uniform demonstration of therapeutic superiority. The strongest evaluations combine an infection-relevant model, an appropriate therapeutic comparator, a measurable antimicrobial or clinical outcome, and evidence that the proposed delivery mechanism contributes to that outcome. A recurring translational limitation is the gap between improved formulation or in vitro antimicrobial performance and demonstrated benefit under clinically relevant conditions.

### 9.2. Preclinical Evidence and Model Relevance

Preclinical evaluation should reflect the biological conditions that influence treatment performance in the intended infection. Simplified planktonic cultures and static biofilm models remain useful for mechanistic screening, although they cannot reproduce all features of clinical infection. Microbial composition, spatial organization, host interactions, fluid movement, nutrient availability, and tissue-specific barriers can influence antimicrobial activity and delivery performance. Positive findings in simplified models should therefore be viewed as initial evidence, not proof of broad translational relevance [132,133].

Current biofilm models vary substantially in their ability to represent clinical infection. Many in vitro systems use single microbial species, simplified growth media, and artificial surfaces and do not reproduce important host factors, tissue structure, fluid dynamics, or polymicrobial interactions. These differences can alter biofilm architecture and antimicrobial susceptibility, limiting the clinical interpretation of positive in vitro findings [134,135]. Translation requires testing strategies that progressively address the biological barriers and exposure conditions relevant to the intended infection instead of relying on findings from a single simplified model.

Model selection should follow the therapeutic question. A system designed for wound infection should be evaluated against barriers relevant to the wound environment. A system intended for implant-associated infection should account for surface colonization, device-associated persistence, and the surrounding tissue environment. Different anatomical sites impose different constraints on drug transport, residence, clearance, and tissue tolerance. Experimental convenience should not determine model selection when the model is poorly matched to the intended indication [133].

A stronger preclinical pathway uses complementary models, with each model addressing a specific uncertainty. Early studies can assess antimicrobial activity, formulation stability, and basic transport behavior. More representative systems can examine penetration and interactions with relevant biological barriers. In vivo studies can then assess exposure, distribution, safety, and therapeutic benefit in the intended setting. Dynamic and tissue-relevant platforms may improve predictive value when they reproduce clinically important barriers more effectively than conventional static systems [132,136].

The purpose of model progression is not simply to increase experimental complexity. It is to reduce uncertainties that could affect clinical performance. Each model should address a specific translational question and provide evidence that the expected therapeutic advantage can persist beyond the original laboratory conditions. Progression should be guided by unanswered questions rather than the routine movement of a formulation through increasingly complex models [132,136].

### 9.3. Exposure, Safety, and Manufacturability

Clinical translation requires more than antimicrobial efficacy. A delivery system must provide predictable exposure, acceptable safety, and reproducible product quality. Post-marketing safety evaluation can provide information that may not be fully captured during preclinical and clinical development. A recent analysis of adverse events associated with omadacycline illustrates the value of pharmacovigilance for identifying safety signals during clinical use [137].

These requirements become especially important when a carrier changes drug distribution, interacts with biological components, persists after drug release, or generates degradation products. In vivo behavior cannot always be predicted from physicochemical characterization because interactions with proteins, cells, and biological barriers can alter the properties of the administered system [136].

Pharmacokinetic evaluation should address the exposure relevant to the therapeutic objective. Depending on the application, this may include local drug concentration, residence time, systemic exposure, clearance, and the duration of pharmacologically active exposure. Measurements of total drug concentration alone may be insufficient when treatment depends on the amount of active drug reaching a specific biological compartment [133].

Manufacturing should be considered during formulation design. A promising laboratory system may be difficult to translate if it depends on poorly controlled assembly, unstable intermediates, complex purification, specialized equipment, or narrow process tolerances. Scale-up can alter critical attributes such as particle characteristics, drug loading, and release behavior. These relationships should be understood early enough to identify the product and process variables that must be controlled to maintain consistent performance [136].

Manufacturing feasibility should be evaluated alongside therapeutic performance. A formulation has credible translational potential only when its critical attributes can be reproducibly controlled while maintaining the exposure and safety profile required for its intended use.

### 9.4. Regulatory and Clinical Translation

The path to clinical use should begin with a defined therapeutic claim. An antimicrobial delivery system may aim to improve treatment of a difficult-to-access infection, reduce systemic toxicity, decrease dosing frequency, improve treatment of persistent infection, or provide another specific clinical advantage. The proposed claim should determine the evidence required to support development. Broad statements of enhanced antimicrobial activity are insufficient when the intended value lies in improved delivery or clinical use [132,133,136].

Translation requires comparison with the relevant therapeutic alternative. The key question is not simply whether a new system performs better than an untreated control. It is whether the additional formulation complexity provides a meaningful advantage over existing therapy. That advantage may involve improved exposure at the target site, reduced toxicity, fewer administrations, greater treatment feasibility, or better clinical outcomes. A complex delivery system that cannot demonstrate such an advantage has a weak translational rationale [138,139].

Regulatory considerations should be incorporated early in development. The composition of the system, its mechanism of action, route of administration, manufacturing process, and interactions with biological or device components can influence the evidence required to establish product quality, safety, and efficacy. Early regulatory planning can help prevent development of a system whose critical attributes cannot be adequately characterized or controlled [130,136].

Overall, translational potential should be judged by the complete therapeutic value of a delivery system. The formulation must address a defined clinical problem and demonstrate a measurable improvement in antimicrobial performance. This benefit should remain evident in biologically relevant models and be supported by acceptable safety and reproducible manufacturing. Additional formulation complexity is justified only when it provides a meaningful advantage over existing treatment options [130,136,138].

## 10. Future Directions and Research Priorities

Future research in antimicrobial drug delivery should move beyond expanding formulation diversity and focus on clearly defined therapeutic gaps. Current global priorities increasingly emphasize the need to align innovation with unmet clinical needs, particularly for severe infections caused by drug-resistant pathogens. Delivery systems should be designed around the requirements of the intended treatment, not simply around the availability of a particular carrier or material. Target product profiles can support this process by defining the patient population, infection type, route of administration, therapeutic objective, and key product characteristics early in development. This approach can help direct research toward systems with a clear clinical role and reduce investment in technologies whose complexity does not provide meaningful therapeutic value [140,141].

### 10.1. From Formulation Screening to Predictive Design

Future development should rely less on empirical formulation screening and place greater emphasis on predictive approaches that link formulation properties with antimicrobial exposure and therapeutic response. Relevant models should integrate drug release and transport with pharmacokinetic and pharmacodynamic behavior. When relevant, they should incorporate biological factors that influence treatment response. Model-informed approaches can combine evidence from different stages of development and help determine earlier whether a proposed system is likely to provide a meaningful pharmacological advantage. Their purpose is not to replace experiments. Instead, they can identify weak candidates, refine promising systems, and focus experimental resources on the most informative questions [142].

Artificial intelligence may further support formulation development by identifying relationships between formulation variables and drug delivery performance. Its usefulness, however, depends on reliable data and experimental validation [143]. Intelligent biomedical systems that combine sensing, decision-making, and therapeutic action represent another emerging area. Recent physically intelligent capsule robots demonstrate how responsive materials can support memory and logic for targeted intervention in vivo, although this technology has not yet been developed specifically for antimicrobial therapy [144]. Model-informed approaches may eventually support more individualized antimicrobial therapy by integrating pharmacokinetic, pharmacodynamic, microbial, and host-related information [142]. Manufacturing considerations should likewise be incorporated early to improve reproducibility and strengthen the translational potential of promising delivery systems [145].

### 10.2. Infection-Relevant Experimental Models

The value of preclinical testing depends on how well a model captures the biological features that influence antimicrobial response in the intended infection. Simplified systems remain useful for early screening. Chronic, device-associated, pulmonary, and other complex infections may require models that represent important features of the local environment. Depending on the research question, these features may include biofilm structure, host tissue, local conditions, microbial interactions, and clinically relevant antimicrobial exposure. Greater complexity does not automatically produce a better model. The model should be no more complex than necessary to answer the research question reliably. A staged approach that progresses from controlled screening systems to models with greater biological relevance can provide a practical balance between experimental efficiency and translational value [134,135].

### 10.3. Comparable and Reproducible Evidence

Future progress will depend on greater comparability between studies. Differences in formulation characterization, antimicrobial testing, infection models, and outcome measures can make it difficult to determine whether apparently different findings reflect true biological effects or methodological variation. Critical experimental conditions and analytical methods should be reported in sufficient detail to permit independent reproduction. Where appropriate, shared outcome measures can support comparisons across studies while preserving flexibility for infection-specific questions. This issue is particularly important in biofilm research, where model selection and experimental conditions can strongly influence the observed antimicrobial response. A clearer distinction between exploratory findings and evidence generated under reproducible conditions would strengthen interpretation across the field [134].

### 10.4. Clinical Relevance as the Primary Criterion for Innovation

Priority should be given to systems that address defined therapeutic problems. These may include inadequate drug exposure at the site of infection, dose-limiting toxicity, limited treatment options against resistant pathogens, or unmet needs in specific patient populations and healthcare settings. Clinical reports and observational studies continue to demonstrate the therapeutic challenges associated with resistant infections, including infections involving *Klebsiella pneumoniae* carbapenemase-producing and other carbapenem-resistant Gram-negative bacteria [146,147].

Global priorities further emphasize that innovation must be accompanied by affordability, accessibility, and practical implementation. The most valuable advances will be those in which pharmaceutical design addresses a clearly defined clinical need and is supported by a realistic path to patient use [140,141,148].

## 11. Conclusions

Advanced antimicrobial drug delivery should be judged by its ability to improve treatment, not by the sophistication of the carrier. Effective therapy requires sufficient antimicrobial exposure at the right site and for the appropriate duration. Achieving this goal can be difficult in infections characterized by biofilms, physiological heterogeneity, and uneven drug distribution. Spatiotemporal adaptation provides a framework for aligning antimicrobial exposure with the location, biological state, and temporal requirements of the therapeutic target. The most promising technologies are those selected according to the specific barrier limiting treatment. Localized depots and controlled-release systems can be valuable when sustained exposure is needed at an accessible infection site. Responsive systems can offer useful control when a reliable biological or external trigger is available. Nanoscale and biological systems may be valuable when transport, penetration, or biological recognition limits treatment. Multifunctional systems can address complex infections when each component provides a clear therapeutic benefit. Important challenges include demonstrating performance in infection-relevant models, establishing reproducible therapeutic benefits, ensuring safety, and maintaining consistent product quality during scale-up and manufacturing. Successful clinical translation requires early definition of the clinical problem, selection of the simplest effective delivery architecture, evaluation in relevant models, and early consideration of manufacturability and practical implementation.

Future progress will depend on connecting formulation design with local drug exposure, biological response, therapeutic benefit, safety, and clinical need. Advanced antimicrobial delivery will have the greatest value when it provides a clear, measurable, and practical advantage for infections that remain difficult to treat with current therapies.

## Figures and Tables

**Figure 1:**
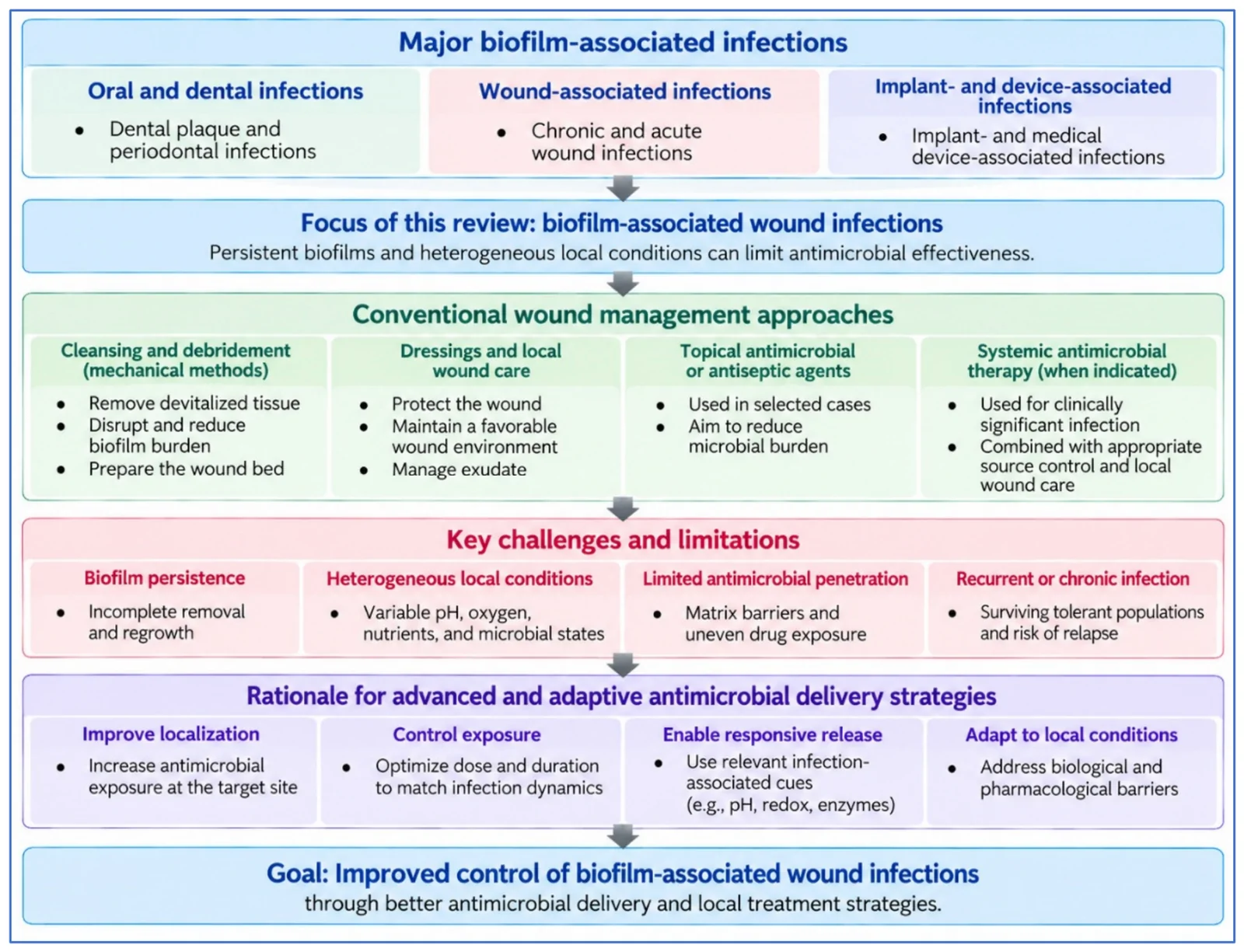
Major biofilm-associated infections, conventional approaches to biofilm-associated wound management, their key limitations, and the rationale for advanced adaptive antimicrobial delivery strategies.

**Figure 2:**
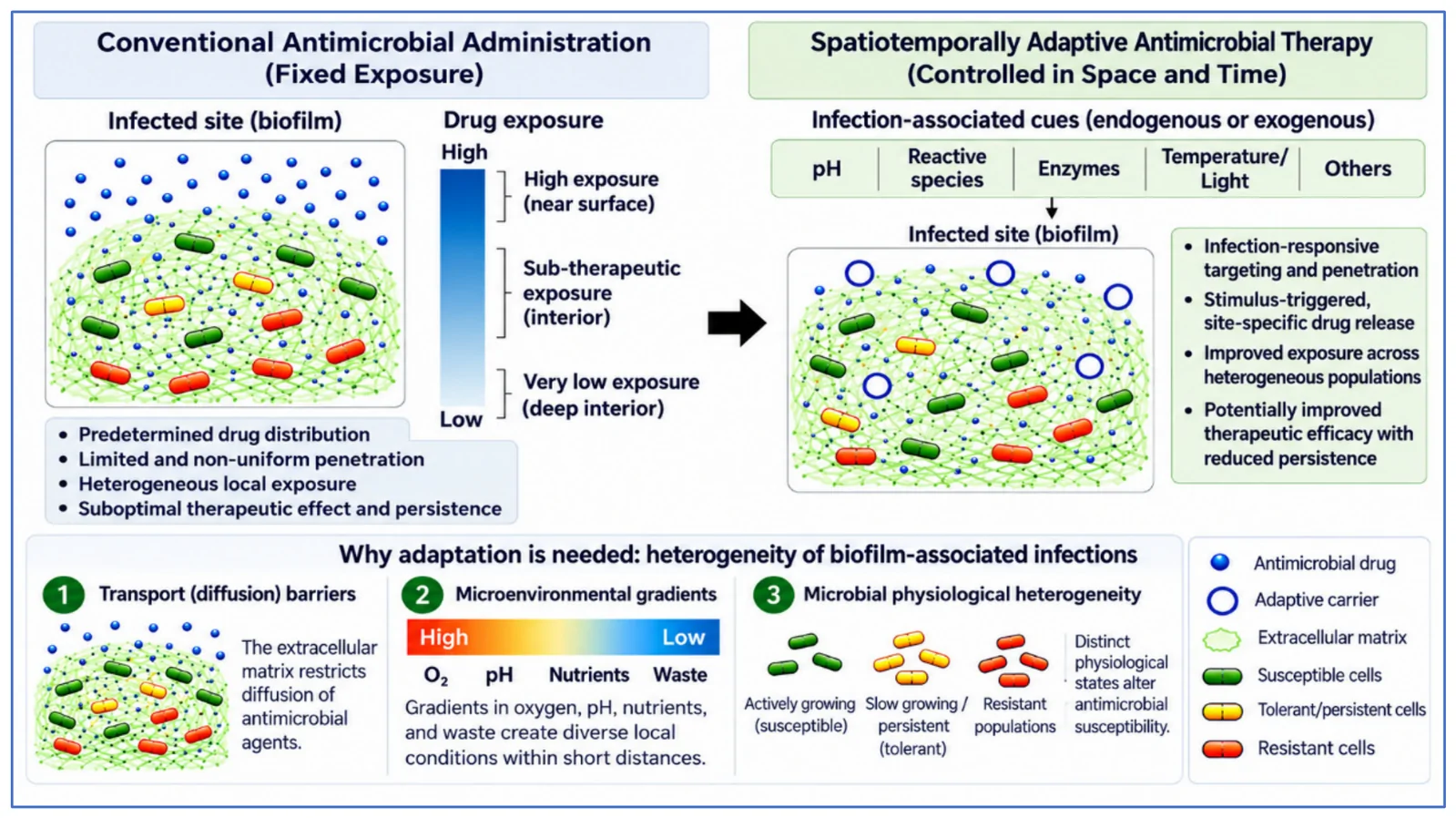
From fixed antimicrobial administration to spatiotemporally adaptive therapy. Spatial variation in drug access and microbial susceptibility can create mismatches between antimicrobial exposure and the biological conditions of infection. Spatiotemporally adaptive delivery aims to improve this match through localized and time-controlled antimicrobial exposure.

**Figure 3:**
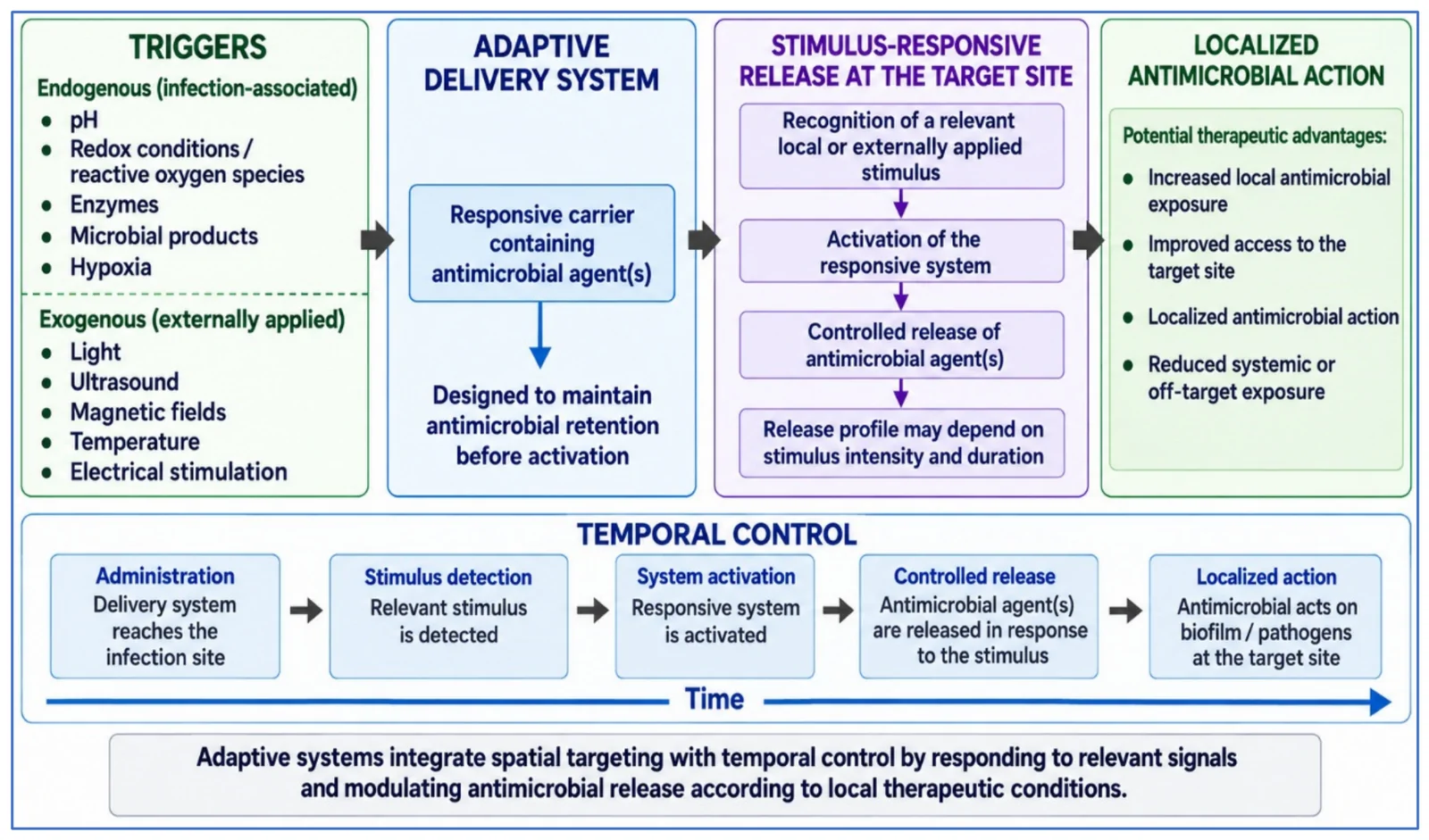
Spatiotemporal adaptation can align antimicrobial exposure with changing conditions at the infection site. Endogenous or externally applied signals activate responsive delivery systems and enable localized, time-controlled antimicrobial exposure. Therapeutic value depends on whether the system improves access, maintains relevant exposure, and addresses a defined limitation within the infected microenvironment.

**Figure 4:**
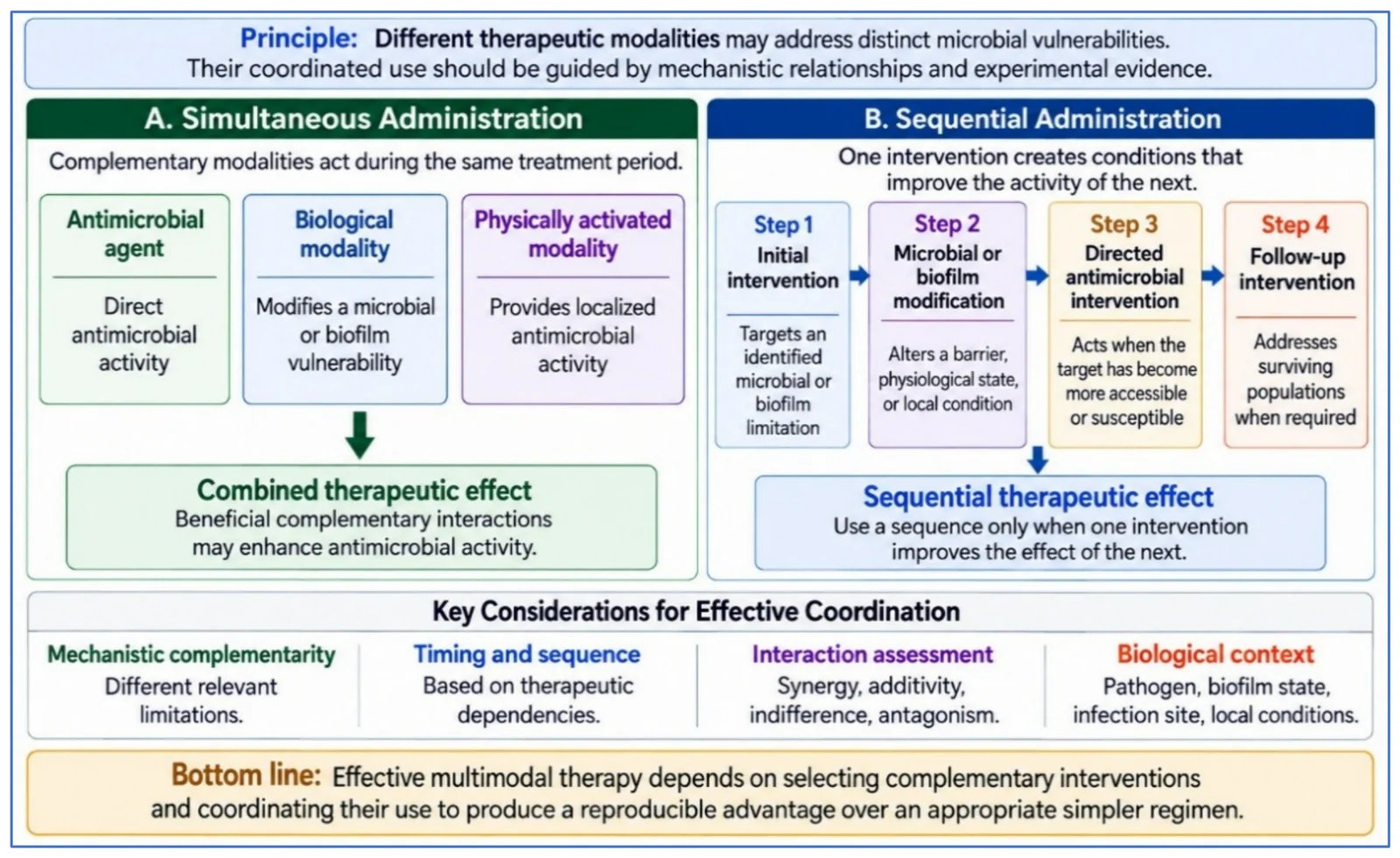
Temporal coordination of multimodal antimicrobial therapy. Complementary interventions may act simultaneously when their functions are compatible or sequentially when one intervention creates conditions that improve the activity of the next. Effective coordination depends on mechanistic complementarity, appropriate timing, interaction assessment, and biological context.

**Table 1:** Dynamic biological barriers that disrupt effective antimicrobial exposure and their consequences for therapy.

Dynamic Biological Barrier	Consequence for Antimicrobial Therapy	Refs.
Spatially heterogeneous drug distribution across infected tissues and compartments	Local concentrations can differ across infected compartments, making systemic exposure an incomplete measure of drug availability at the site of infection.	[26,29]
Restricted intrabiofilm transport within structured microbial communities	Matrix interactions, microbial architecture, and local diffusion constraints can alter the rate and extent of antimicrobial access to different biofilm regions.	[26,28]
Temporal evolution of the infection microenvironment	Infection progression, inflammation, tissue injury, and chronicity can change the biological conditions under which antimicrobial exposure occurs.	[30,31,33]
Physiological heterogeneity within microbial populations	Differences in metabolic activity, growth, and stress responses can produce variable susceptibility among neighboring cells exposed to the same antimicrobial.	[7,8]
Dormancy and persister survival	Transiently inactive or persister subpopulations can survive treatment and contribute to regrowth after antimicrobial concentrations decline.	[27,35]
Uneven or insufficient antimicrobial exposure	Survival under variable pharmacological conditions can maintain populations under selection pressure and contribute to conditions associated with resistance emergence.	[26,28]

**Table 2:** Triggering mechanisms for adaptive antimicrobial systems: signal source, activation mechanism, therapeutic value, major limitation, and representative references.

Trigger	Signal Source	Activation Mechanism	Therapeutic Value	Major Limitation	Refs.
pH	Altered local acidity	Protonation or acid-sensitive bond cleavage changes the responsive state	Links activation to acidic conditions	pH varies across infection sites	[16]
Redox conditions/ ROS	Altered redox conditions and/or ROS levels	Redox-sensitive groups undergo oxidation, reduction, or cleavage	Responds to local redox imbalance	Similar conditions occur outside infection	[16]
Enzymes	Microbial or infection-associated enzymatic activity	Enzyme-mediated substrate cleavage initiates activation	Couples response to specific biochemical activity	Enzyme expression varies between pathogens and sites	[16,21]
Microbial secretions	Secreted microbial products	Recognition or chemical interaction with the signal initiates activation	Connects activation directly to microbial activity	Signal production depends on microbial state	[16]
Hypoxia	Reduced oxygen availability within the infected microenvironment	Hypoxia-responsive chemical groups alter the release behavior in response to bacterial-induced oxygen depletion	Couples antimicrobial release to the severity of local hypoxia	Hypoxia may vary spatially and temporally and is not unique to infection	[62,63]
Light	Applied optical energy	Photochemical or photothermal conversion initiates the response	Provides precise temporal initiation	Limited penetration restricts deeper targets	[16,18]
Ultrasound	Applied acoustic energy	Acoustic stimulation can induce mechanical effects, cavitation, localized heating, or system-dependent phase transitions	Provides externally controlled activation and can reach targets beyond the penetration range of optical irradiation	Response depends on acoustic parameters, tissue properties, and the ultrasound-responsive mechanism	[60]
Magnetic field	Applied magnetic field	Magnetic fields can induce motion, field-directed localization, or heating	Enables remote stimulation	Requires a magnetically responsive component	[16]
Temperature	Applied or locally generated heat	Thermal transition alters the responsive state	Enables threshold-dependent activation	Local heating must be controlled	[16]
Electrical stimulation	Applied electrical energy	Electrical input produces an electrochemical or physicochemical change	Rapid, adjustable external activation	Requires practical and safe energy delivery	[21]
Multiple triggers	Two or more biological and/or physical signals	Inputs act jointly or sequentially	Integrates complementary activation cues	Requires coordinated responses to more than one input	[16,18]

**Table 3:** Critical comparison of therapeutic architectures for spatiotemporal antimicrobial adaptation.

Therapeutic Architecture	Localization	Access	Responsiveness	Temporal Control	Persistence	Key Limitation	Clinical Status and Future Potential	Refs.
**Nanoscale carriers**	Design-dependent	Enhanced transport potential	Endogenous/external	Tunable	Variable	Safety and scale-up	Mostly preclinical; promising for improved transport	[79,83]
**Localized and depot systems**	Strong local retention	Diffusion-limited	Incorporable	Sustained/triggered	High	Limited deep access	Strong potential for localized infections	[17,89]
**Surface-associated systems**	Treated interface	Adjacent microorganisms	Coating-dependent	Sustained	High locally	Limited spatial reach	Relevant to device-associated applications	[81,96]
**Biomimetic and biological systems**	Interaction-dependent	System-dependent	Biological	Mechanism-dependent	Variable	Standardization and stability	Early-stage translational potential	[82,102,104]
**Multifunctional architectures**	Multi-component	Multiple barriers	Multiple triggers	Coordinated/sequential	Component-dependent	High development complexity	Promising for complex infections	[86,87,88]

## Data Availability

No new data were created or analyzed in this study. Data sharing is not applicable to this article.
